# Rational Treatment of Decomposing Pulps

**Published:** 1904-12-15

**Authors:** J. P. Buckley


### RATIONAL TREATMENT OF DECOMPOSING PULPS.

BY J. P. BUCKLEY, D.D.S.

In selecting drugs to be used in the rational treatment of these conditions, I would eliminate the question of coagulation and select agents with reference only to their ability to unite chemically with the end-products, resulting from pulp decomposition. In this connection we should remember that the putrescent condition has been brought about through the agency of micro-organisms by a gradual analytic process; that many of these germs are pathogenic in character and that among the first products of importance are hydrogen sulphide, putrescin, cadaverin and neuridin. The last-named compound, being non-infectious, is of little importance, other than to know that it is a nitrogenous substance from which ammonia is evolved by further putrefaction. Still, according to Vaughan and Novy, while pure neuridin is non-poisonous, it posesses a toxic action as long as it is contaminated with other poisonous products of putrefaction. This holds true for all non-poisonous bases. Hydrogen sulphide is important because it is an *acid gas* with a disagreeable odor, having local irritant properties; and also because of the part it plays in the discoloration of the tooth structure. However, I must say here, while I realize that hydrogen sulphide is an active chemical agent that, in my opinion, it has been greatly over-estimated in the role it assumes in the discoloration of teeth from the decomposition of the pulp tissue. Putrescin and cadaverin are perhaps the most important compounds, in so far as the correction of the putrescent condition is concerned, known to be formed in the splitting up of the proteid molecule. Like neuridin, they are basic nitrogenous compounds, capable of undergoing further putrefaction, evolving ammonia; but, unlike this compound, while they were at first regarded as physiologically inactive, both these bases have been proven, by Scheurlen, Grawitz and others, to be capable of producing inflammation and necrosis.

Among the gases produced, then, in pulp decomposition are carbon dioxide, ammonia and hydrogen sulphide. As these gases are evolved in those cases where there is no free exit from the pulp chamber, through a cavity, pressure is produced and in many instances they escape through the apices of the roots, carrying the poisonous ptomaines into the surrounding tissues; inflammation is thereby produced, and alveolar abscess established.

In those cases where we open into the pulp chamber and find a pulp undergoing the process of decomposition, when the ptomaines and end-products have not been forced through into the apical tissue, our treatment should be to at once *hermetically* seal into the pulp chamber an agent which is volatile and thereby penetrating, and which, as it comes in contact with the end-products, will unite chemically, converting them into odorless and non-infectious compounds. Such an agent we have in *formaldehyde*, a gas, $CH_2O$, which occurs in commerce as a forty percent aqueous solution known as *formalin*.

It has long since been known that ammonia is one of the chief end-products in the splitting up of the proteid molecule. It is also known that formaldehyde unites with ammonia to form a solid compound, which is odorless, colorless and has a sweetish taste, known commercially as urotropin, chemically as hexamethylene-tetramin, with a chemical formula $(CH_2)_6N_4$. It is stated also on good authority that formaldehyde unites chemically with hydrogen sulphide, and basic ptomaines, forming inodorous compounds. Formalin, however, is too strong a solution for our general use; therefore, believing that the facts remain practically unchanged in the process of decomposition, I have been using cresols, in combination with formalin, which act chemically upon the fatty constituents. Cresols are homologs of carbolic acid. There are three—meta-cresol, ortho-cresol, and para-cresol. The product best suited for our use is *tri-cresol*, a refined mixture of the three. It is a nearly colorless liquid, of a creosote-like odor, and is soluble in water to the extent of a five percent solution. Tri-cresol has been selected as the vehicle with which to dilute formalin for three reasons:

1. It is miscible with formalin in all proportions, thus making a good pharmacal product.

2. It is a good germicide—nearly three times as powerful as carbolic acid.

3. It acts chemically upon the fatty constituents, thereby properly disposing of these substances.

The formula which I have been using with gratifying results in the treatment of putrescent pulps is:

R

Tri-cresol,
Formalin, aa fl. dr. j (4.0 cc.)

M. Sig. On a small pledget of cotton, hermetically seal in the pulp chamber from twenty-four to forty-eight hours. One treatment is generally sufficient.

Treatment of abscess without Fistules.—In the treatment of abscesses without a fistulous opening, it is well to modify this formula. In these cases the decomposition of the pulp tissue is complete. The intermediate products (ptomaines) have largely been broken up, pus has been formed from the tissue surrounding the end of the roots, and the first step in treating such an abscess is to mechanically evacuate the pus. We have no necessity for using formaldehyde then in the same strength solution as in those cases where the pulp chamber, root canals and tubuli are filled with the putrescent material. The point I desire to impress is that formaldehyde in this strength solution *must* be confined to the tooth structure, for it is one of the most irritating agents known to the therapeutist. A safe formula for abscesses without a fistula is:

R

| | |
|---|---|
| Tri-cresol, | fl. dr. j (4.0 cc.) |
| Formalin, | fl. dr. ss (2.0 cc.). |

M. Sig. Mechanically evacuate the pus and, on cotton, hermetically seal in the canals from twenty-four to forty eight hours. Two or three, often times one treatment is sufficient.

Tri-cresol, besides its germicidal power, dissolves the fatty globules, which, if treated with alcohol (as is usually done in drying the canal), produces *lysol*, a good antiseptic. It is plain to be seen, then, that the *poisonous gases* and *liquids* resulting from pulp decomposition are converted by the proper use of formaldehyde and tri-cresol into *non-poisonous liquids* and *solids*, which are themselves antiseptic and germicidal in character. How can we imagine a more thorough sterilization of the dentine than to chemically produce within this tubular structure substances which possess these properties?

Thus I feel justified in speaking of the use of this remedy as a *rational treatment* for the conditions under consideration. Marvelous results are obtained, and at the same time we know why.

It should be remembered that the formulas given are for general use. The practical dentist will soon find that it may be best to vary the proportion of the ingredients of the mixture according to the case at hand. One great advantage in using remedies containing formaldehyde is that the medicament *must be hermetically sealed* in the tooth in order to obtain the best results. This prevents the saliva from contaminating the medicine within the tooth, and the medicine from contaminating the saliva in the patient's mouth.

The use of neither formaldehyde nor tri-cresol alone is original with the writer. I have studied the action of drugs, trying to place the treatment of these conditions upon a rational basis, and am gratified to know that such has been accomplished by the combination of formalin and tri-cresol in the manner which I have suggested.

In conclusion, I desire to say that many of the statements made in this paper are based upon well established chemical facts. Therefore, I cannot lay much claim to originality. However, I have endeavored to make a correct application of the chemical principles involved. The theory in regard to the decomposition of the pulp tissue may seem, perhaps, not to have been scientifically demonstrated; but it is the result of my laboratory investigation and also is in harmony with my close clinical observation. As such, I present it to you for your consideration.—*Items.*

